# Untargeted Metabolomic Analysis of Amniotic Fluid in the Prediction of Preterm Delivery and Bronchopulmonary Dysplasia

**DOI:** 10.1371/journal.pone.0164211

**Published:** 2016-10-18

**Authors:** Eugenio Baraldi, Giuseppe Giordano, Matteo Stocchero, Laura Moschino, Patrizia Zaramella, Maria Rosa Tran, Silvia Carraro, Roberto Romero, Maria Teresa Gervasi

**Affiliations:** 1 Department of Women’s and Children’s Health, University of Padova, Padova, Italy; 2 Città della Speranza Institute of Pediatric Research (IRP), Padova, Italy; 3 S-IN Soluzioni Informatiche, Vicenza, Italy; 4 Perinatology Research Branch, NICHD, NIH, DHHS, Wayne State University/Hutzel Women's Hospital, Detroit, United States of America; National Research Council of Italy, ITALY

## Abstract

**Objective:**

Bronchopulmonary dysplasia (BPD) is a serious complication associated with preterm birth. A growing body of evidence suggests a role for prenatal factors in its pathogenesis. Metabolomics allows simultaneous characterization of low molecular weight compounds and may provide a picture of such a complex condition. The aim of this study was to evaluate whether an unbiased metabolomic analysis of amniotic fluid (AF) can be used to investigate the risk of spontaneous preterm delivery (PTD) and BPD development in the offspring.

**Study design:**

We conducted an exploratory study on 32 infants born from mothers who had undergone an amniocentesis between 21 and 28 gestational weeks because of spontaneous preterm labor with intact membranes. The AF samples underwent untargeted metabolomic analysis using mass spectrometry combined with ultra-performance liquid chromatography. The data obtained were analyzed using multivariate and univariate statistical data analysis tools.

**Results:**

Orthogonally Constrained Projection to Latent Structures-Discriminant Analysis (oCPLS2-DA) excluded effects on data modelling of crucial clinical variables. oCPLS2-DA was able to find unique differences in select metabolites between term (n = 11) and preterm (n = 13) deliveries (negative ionization data set: R^2^ = 0.47, mean AUC ROC in prediction = 0.65; positive ionization data set: R^2^ = 0.47, mean AUC ROC in prediction = 0.70), and between PTD followed by the development of BPD (n = 10), and PTD without BPD (n = 11) (negative data set: R^2^ = 0.48, mean AUC ROC in prediction = 0.73; positive data set: R^2^ = 0.55, mean AUC ROC in prediction = 0.71).

**Conclusions:**

This study suggests that amniotic fluid metabolic profiling may be promising for identifying spontaneous preterm birth and fetuses at risk for developing BPD. These findings support the hypothesis that some prenatal metabolic dysregulations may play a key role in the pathogenesis of PTD and the development of BPD.

## Introduction

Preterm delivery (PTD) is a major challenge in the field of obstetrics and neonatology. Since 2006 preterm birth rates have been declining both in the United States and in European countries. Nevertheless, prematurity remains a major cause of morbidity and mortality worldwide, which exceed those of infants born full-term[1,2]. Preterm neonates are at increased risk of both short- and long-term pathological outcomes[3–6] and, among these, bronchopulmonary dysplasia (BPD) accounts for the vast majority of cases of chronic lung disease after premature birth[7]. In a recent workshop sponsored by the National Heart, Lung, and Blood Institute (NHLBI) on the primary prevention of chronic lung diseases, participants agreed that the insults leading to BPD may begin in-utero, and operate through gene-environment interactions and epigenetic mechanisms[8]. Such early insults may operate by altering the trajectory of airway growth and development in these children with effects persisting into adulthood[4,7–9].

The pathogenesis and link between spontaneous preterm delivery and BPD is poorly understood. Much of the progress made in the understanding of the causes of preterm labor and BPD has derived from hypothesis-driven research[3,10–13]. Although this approach has yielded important information, we propose that using a hypothesis-free approach based on high-throughput analytical techniques has the potential to provide a more comprehensive description of the complex mechanisms and interactions behind these disorders[14]. Amniotic Fluid (AF) is an ideal matrix for characterizing maternal-fetal conditions and contains fetal lung fluid. AF is rich in low molecular weight metabolites, and this makes it an appropriate biological matrix for the application of metabolomics [15,16].

The aim of this exploratory study was to evaluate whether the untargeted metabolic profiling of AF in women with symptoms of preterm labor can be useful to investigate the risk of spontaneous preterm birth and BPD development in the offspring.

## Materials and Methods

### Study design and population

We conducted a study on 32 infants born from 32 mothers who had undergone an amniocentesis between 21 and 28 gestational weeks because of spontaneous preterm labor with intact membranes (due to PROM, chorioamnionitis, flow alterations or other causes). Amniocentesis had been performed at the participating institutions (Padova and Treviso general hospitals, Veneto region, Italy) to assess the microbial state of the amniotic cavity and to diagnose intra-amniotic infection/inflammation[17]. Twins and newborns with congenital anomalies were excluded. The amniotic fluid samples were collected by the same physician (MTG) with a standardized procedure.

Twenty-four of the 32 AF samples were obtained from trans-abdominal amniocentesis, the other 8 by amniocentesis at the time of cesarean delivery. Five milliliters of AF were collected, frozen and stored at—80° until the time of the analysis. For the purposes of our data analysis the samples were considered as follows:

1. In the first step, we aimed to assess whether preterm delivery could be discriminated by the metabolomic profile of amniotic fluid, by focusing on samples (n = 24/32) collected at least 1 day before birth. Among these, the metabolomic profile was compared with those of patients delivered preterm (PTD group, n = 13/24) and those of term newborns (TD group, n = 11/24).
2. The second step of the study consisted of determining whether amniotic fluid analysis could discriminate infants bound to develop BPD. Twenty-one samples from pregnancies which resulted in a preterm delivery (n = 21/32) were analyzed according to whether infants developed BPD (PTD with BPD, n = 10/21; PTD with no BPD, n = 11/21).

The study was approved by the Institutional Review Boards of the participating Institutions (Comitato Etico per la Sperimentazione, Padova and Treviso General Hospitals, protocol number 24139, Veneto Region, Italy). All the women gave their written informed consent to their AF being used for research purposes.

### Clinical definitions

Spontaneous preterm labor was defined by the presence of regular uterine contractions associated with cervical changes occurred before 37 completed weeks of gestation and requiring hospitalization[2]. Clinical chorioamnionitis was defined as the presence of maternal fever, maternal and/or fetal tachycardia, elevated maternal CRP, uterine fundal tenderness, and purulent or foul-smelling amniotic fluid[18]. PTD was defined as birth before 37 weeks of gestation. Infants were followed until 3 months of life and BPD was defined as the need for supplemental oxygen at 36 weeks’ postmenstrual age [19].

### Amniotic fluid metabolite analysis by Mass Spectrometry (MS) combined with Ultra- Performance Liquid Chromatography (UPLC)

#### Sample preparation

The analysis was performed at the Mass Spectrometry Laboratory of the Department of Women’s and Children’s Health at the Città della Speranza Institute of Pediatric Research (IRP) (Padova University and General Hospital). All the procedures involved in the preparation of the AF samples are described in the S1 Material.

#### Chromatographic analysis and mass spectrometry

**Fig 1:**
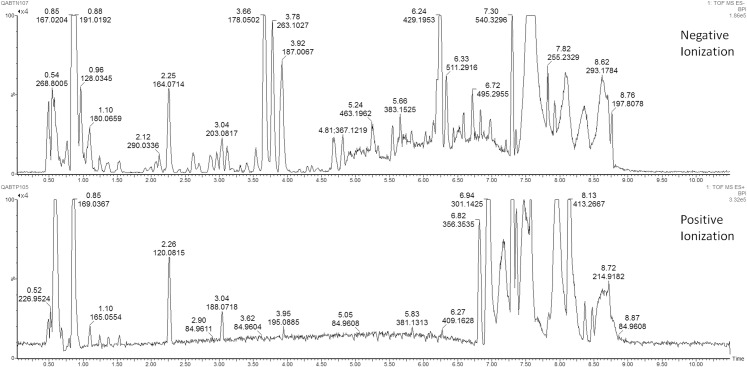
Chromatographic profiles of an amniotic fluid sample, derived from the reverse-phase column HSS T3, with both positive and negative ionization mode.

The metabolic analysis of the AF samples was performed with a Q-ToF Synapt G2 (Waters) high resolution mass spectrometer interfaced with a UPLC (Ultra Performance Liquid Chromatography) system (Acquity-Waters), characterized by high chromatographic resolution, short analytical time and enhanced sensitivity. The chromatographic analysis was performed through the reverse-phase HSS T3 column (Acquity HSS T3, Waters co., Miliford, MA USA) at 40°C. MS analysis was conducted with an Electrospray source (ESI) in both positive and negative ionization mode. Fig 1 shows chromatographic profiles of an amniotic fluid sample. A detailed description of chromatographic analysis, processing and pre-treatment of data is reported in the S1 Material.

### Statistical data analysis

After a preliminary exploratory data analysis on the clinical data (metadata) using Principal Component Analysis (PCA) and Projection to Latent Structures-Discriminant Analysis (PLS-DA) to exclude any confounding effects with respect to the clinical groups under investigation, we applied a new version of PLS-DA called orthogonally Constrained PLS-DA (oCPLS2-DA)[20] to the data sets generated by UPLC-MS that enables orthogonal constraints to be included in the latent variable calculation. A description of the method is provided in the S1 Material. The main advantage to use oCPLS2-DA in data modelling consists in the possibility to remove the effects of potential factors that can influence the calculation of the latent variables by projection. Specifically, the latent structure discovered by oCPLS2-DA results to be orthogonal to the factors used as constraints. In our study, the constraints were defined on the basis of the metadata in order to obtain models were the variation of the metabolite content of the collected samples is explained only by latent variables that are independent from the metadata. To simplify the model’s interpretation, we applied a post-transfomation of the oCPLS2-DA model[21]. To avoid over-fitting and prove the robustness of the models obtained, we performed *N*-fold full cross-validation with different values of *N* (*N* = 6,7,8) and permutation tests on the class responses (500 random permutations) in accordance with good practice for model validation [22]. The results of the cross-validation procedure were expressed as Q^2^, while those of the permutation test as p-values. In addition, stability selection based on Monte-Carlo sampling was applied [23]. Specifically, 200 subsets were extracted from the collected samples by Monte-Carlo sampling (with prior probability of 0.70) and used to build independent oCPLS2-DA models. The performance in prediction of each model was estimated by ROC analysis of the outcomes obtained by predicting the samples which had been excluded during subsampling. The first 50 variables having the highest regression coefficients were selected for each independent oCPLS2-DA model. The variables selected for more than the 90% of all the models were investigated as putative markers and submitted to a t-test and ROC (Receiver Operating Characteristics) curve analysis. Since multivariate data analysis explores the correlation structure of the collected data while univariate data analysis investigates the properties of single variables, we also performed the latter using t-test with false discovery rate correction and ROC analysis in order to complement the results of the multivariate data analysis. The PCA and PLS-DA were performed using SIMCA 13 (Umetrics, Umea, Sweden). The R 3.0.2 platform (R Foundation for Statistical Computing) was used for univariate data analysis (t-test with false discovery rate correction and ROC analysis[24]), and user-written R functions enabled us to run the oCPLS2-DA and the post-transformation of the discriminant models.

### Identification of relevant variables

To identify the relevant variables characteristic of each clinical group and emerging from the correlation loading plot, we searched the main available metabolome databases (Human Metabolome DataBase (HMDB) and METLIN), which enable comparison to be drawn between the spectroscopic characteristics of the variables and those of known metabolites. This is the first in a series of different steps that ultimately lead to the identification of potential key metabolites, and it enables both the chemical structure and the biological activity of the putative molecules to be hypothesized. More information on the parameters used for the identification of key metabolites are provided in the S1 Material.

## Results

Table 1 displays the demographic and clinical characteristics of the infants included in the first and second steps of our data analysis, together with details regarding the mothers and their gestations. In 9 cases belonging to the ‘PTD with BPD’ group and in 8 belonging to the ‘PTD without BPD’ group, placental pathology was available. In these groups acute histologic chorioamnionitis was detected in 3 and 4 cases, respectively.

**Table 1:** Demographic and clinical characteristics for all the groups analyzed in our study.

	PTL with PTD Group vs PTL with TD Group		PTD with BPD Group vs PTD with no BPD Group	
	PTD Group (n = 13)	TD Group (n = 11)	PTD with BPD Group (n = 10)	PTD with no BPD Group (n = 11)
**GA at amniocentesis (wks)**	24 (23–26)	26 (23–26)	26 (23–26)	26 (24–27)
**GA at delivery (wks)**	27 (26–31)	39 (38–39)	26 (26–26)	27 (26–34)
**Amniocentesis-to-Delivery interval (days)**	23 (7–49)	98 (83–114)	0 (0–16)	24 (1–53)
**Males**	7	8	7	7
**BW (gr)**	1035 (900–2075)	3595 (3270–3685)	786 (635–900)	1260 (925–2500)
**Cesarean Section**	4	1	6	4
**Maternal Age at amniocentesis (yrs)**	29 (27–31)	32 (31–36)	30.5 (24–39)	30 (29–34)
**Maternal BMI**	22 (21–24)	20 (19.9–23.6)	25.7 (20.2–25.7)	22 (20–24.38)
**Smoke during pregnancy**	0	1	0	0
**Maternal Ethnicity**	1 African; 1Asian; 11 Caucasian	1 African; 10 Caucasian	1 African; 9 Caucasian	1 Asian; 10 Caucasian
**Previous miscarriages**	4	4	4	2
**Trans-abdominal amniocentesis sampling**	13	11	4	9
**Maternal therapy at amniocentesis**	9 Betamethasone; 6 Atosiban; 1 Progesterone; 1 Nifedipine	7 Betamethasone; 4 Atosiban; 3 Progesterone; 1 Nifedipine	6 Betamethasone; 3 Atosiban; 1 Progesterone; 2 Nifedipine	8 Betametasone; 8 Atosiban; 1 Progesterone; 1 Nefedipine

Data are expressed as median (IQR) or as absolute number of subjects. PTD = preterm delivery; TD = term delivery; PTL = preterm labor; BPD = bronchopulmonary dysplasia; GA = gestational age; BW = birth weight; SGA = small for gestational age; BMI = body mass index.

### Amniotic fluid metabolome in women who delivered preterm vs. those who delivered at term

**Fig 2:**
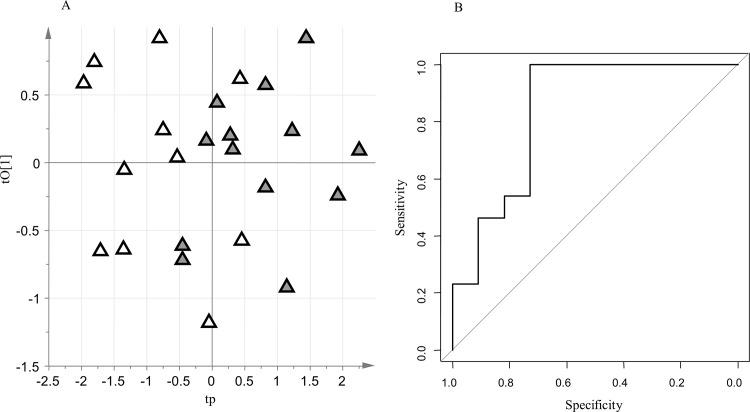
oCPLS2-DA model for PTD group versus TD group (negative data set); A: score scatter plot after post-transformation of the model (PTD are reported as grey triangles while TD as open triangles); B: ROC curve of the model, calculated by 7-folds full cross-validation.

Among the samples collected at least 1 day before delivery, those associated with PTD (n = 13) were compared with those associated with term delivery (TD, n = 11). The negative data set included 1369 RT_mass variables, while the positive data set included 1742 RT_mass variables. The following metadata were considered: maternal age at amniocentesis, maternal BMI, previous miscarriages, maternal therapy at amniocentesis (nifedipine, betamethasone, atosiban, progesterone), gestational age at amniocentesis and sex of newborn. The PCA model on the metadata did not reveal clusters corresponding to the two groups under investigation. The PLS-DA models constructed considering the metadata as the X-block were also unreliable in modeling the differences between the two groups. There was therefore no confounding effect between the metadata and the clinical groups. Reliable oCPLS2-DA models were built to explore the structured variation in the negative data set (A = 1+2 components, R^2^ = 0.47, Q^2^_6-folds_ = 0.25, Q^2^_7-folds_ = 0.26, Q^2^_8-folds_ = 0.31, p-value permutation test for Q^2^_7-folds_ = 0.044, area under curve for ROC analysis at the 95% confidence level estimated by 7-fold full cross-validation = 0.66–1.00, specificity estimated by 7-fold full cross-validation = 0.73, sensitivity estimated by 7-fold full cross-validation = 1.00. ROC analysis for predicted outcomes: mean area under curve = 0.65, mean specificity = 0.70, mean sensitivity = 0.79; Fig 2), and in the positive data set (A = 1+2 components, R^2^ = 0.47, Q^2^_6-folds_ = 0.38, Q^2^_7-folds_ = 0.35, Q^2^_8-folds_ = 0.37, p-value permutation test for Q^2^_7-folds_ = 0.026, area under curve for ROC analysis at the 95% confidence level estimated by 7-fold full cross-validation = 0.67–1.00, specificity estimated by 7-fold full cross-validation = 0.82, sensitivity estimated by 7-fold full cross-validation = 0.85. ROC analysis for predicted outcomes: mean area under curve = 0.70, mean specificity = 0.83, mean sensitivity = 0.72; S1 Material: S1 Fig). Stability selection based on Monte-Carlo sampling enabled us to select a subset of 21 promising key metabolites. Univariate data analysis based on the t-test with false discovery rate correction (q-value threshold equal to 20%) and ROC analysis did not provide any additional features of interest.

By searching the available online metabolite databases and studying the fragmentation spectra, we were able to identify a subset of biochemicals that underpin the models we have found (Table 2). The PTD group was characterized by higher levels of variables attributable to the following classes of compounds: amino acids and their derivatives, unsaturated hydroxy fatty acids (putative metabolite: 3-methoxybenzenepropanoic acid), oxylipins (putative metabolite: 4-hydroxy nonenal alkyne), fatty aldehydes (putative metabolite: muconic dialdehyde). On the other hand, the TD group was characterized by higher levels of variables related to phosphatidylcholine.

**Table 2:** Key metabolites emerged from the search on the metabolome databases: PTD Group vs TD Group.

Group	RT	m/z	Adduct	Mass error (ppm)	AUC ROC (CI 95%)	Sp	Se	Putative metabolites or Metabolite class	Fragmentation pattern
PTD Group	2.23	109.0292	[M-H]-	2	0.55–0.99	0.70	0.82	Muconic dialdehyde	No fragment
	5.06	197.0808	[M-H]-	2.5	0.60–1.00	0.70	0.82	Dicarboxylic unsaturated fatty acid	153.0921, 135.0815, 111.0451
	6.63	421.1606	[M-H]-	7	0.63–1.00	0.70	0.82	Amino acid chain	No fragment
	5.06	179.0699	[M-H]-	8	0.59–0.97	0.60	0.91	3-Methoxybenzenepropanoic acid	No fragment
	5.05	153.092	[M+H]+	6	0.67–1.00	0.70	0.82	4-hydroxynonenal alkyne	No fragment
	0.76	128.0711	[M+H]+	3	0.53–0.96	0.80	0.64	Hydropyridine	No fragment
TD Group	5.28	744.5888	[M+H]+	1	0.61–1.00	1.00	0.64	Phosphatidylcholine	No fragment

RT = Retention Time; m/z = mass-to-charge ratio; ppm = part per million; AUC = Area under the Curve; ROC = receiver operating characteristics; Sp = specificity; Se = sensitivity; the optimal threshold used for specificity and sensitivity is the point closest to the top-left part of the plot with perfect sensitivity or specificity.

To avoid the possible confounding effect of metabolic processes closely related to delivery, the comparison between PTD and TD was also performed including only the samples collected at least 5 days prior to delivery (10 PTD and 11 TD). The results were similar (data not shown).

### Amniotic fluid metabolome and subsequent risk of BPD development

**Fig 3:**
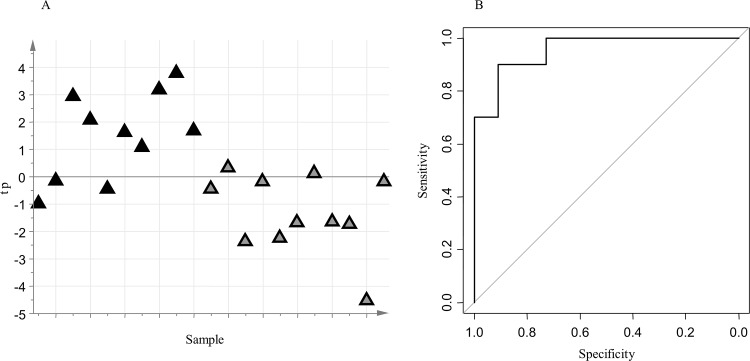
oCPLS2-DA model for PTD with BPD versus PTD without BPD (negative data set); A: score scatter plot of the model (PTD with BPD are reported as black triangles, PTD without BPD as grey triangles); B: ROC curve of the model, calculated by 7-folds full cross-validation.

Considering only the subjects delivered preterm, a comparison between those who developed BPD (n = 10) and those who did not (n = 11) was undertaken. The negative data set included 1384 RT_mass variables, while the positive data set included 1826 RT_mass variables. The following metadata were considered: maternal age at amniocentesis, maternal BMI, previous miscarriages, maternal therapy at amniocentesis (nifedipine, betamethasone, atosiban, progesterone), gestational age at amniocentesis, sex of newborn and trans-abdominal amniocentesis method. The analysis of the metadata by PCA and PLS-DA excluded significant confounding effects between clinical groups and metadata. Reliable oCPLS2-DA models were built to explore the structured variation in the negative data set (A = 1 component, R^2^ = 0.48, Q^2^_6-folds_ = 0.36, Q^2^_7-folds_ = 0.43, Q^2^_8-folds_ = 0.42, p-value permutation test for Q^2^_7-folds_ = 0.004, area under curve for ROC analysis at the 95% confidence level estimated by 7-fold full cross-validation = 0.89–1.00, specificity estimated by 7-fold full cross-validation = 0.91, sensitivity estimated by 7-fold full cross-validation = 0.90. ROC analysis for predicted outcomes: mean area under curve = 0.73, mean specificity = 0.81, mean sensitivity = 0.84; Fig 3), and in the positive data set (A = 1+1 components, R^2^ = 0.55, Q^2^_6-folds_ = 0.36, Q^2^_7-folds_ = 0.38, Q^2^_8-folds_ = 0.42, p-value permutation test for Q^2^_7-folds_ = 0.022, area under curve for ROC analysis at the 95% confidence level estimated by 7-fold full cross-validation = 0.74–1.00, specificity estimated by 7-fold full cross-validation = 0.70, sensitivity estimated by 7-fold full cross-validation = 0.91. ROC analysis for predicted outcomes: mean area under curve = 0.71, mean specificity = 0.78, mean sensitivity = 0.79; S1 Material: S2 Fig). For both models, the predictive latent variable resulted to be not correlated with the presence of chorioamnionitis. Stability selection based on Monte-Carlos sampling enabled the selection of a subset of 19 key metabolites. Univariate data analysis based on the t-test with false discovery rate correction (q-value threshold equal to 20%) and ROC analysis produced no additional interesting features.

Searching the available online metabolite databases and studying the fragmentation spectra enabled us to identify a subset of putative metabolites (Table 3). The PTD group with BPD featured higher levels of leucinic acid, hydroxy fatty acids (putative metabolite: 4-Hydroxy-3-methylbenzoic acid and 2-hydroxy caprylic acid), oxy fatty acids (putative metabolite: 3-oxo-dodecanoic acid), and a metabolite ascribable to a sulphated steroid. Compared to the BPD PTD group, the group without BPD was characterized by higher levels of S-Adenosylmethionine and aminoacid chains and 3b,16a-Dihydroxyandrostenone sulfate (DHEAS).

**Table 3:** Key metabolites emerged from the search on the metabolome databases: PTD with BPD Group vs PTD with no BPD Group.

Group	RT	m/z	Adduct	Mass error (ppm)	AUC ROC (CI 95%)	Sp	Se	Putative metabolites or Metabolite class	Fragmentation pattern
PTD with BPD	3.99	131.0706	[M-H]-	5	0.53–0.97	0.91	0.60	Leucinic acid	85.0653
	4.02	151.0393	[M-H]-	5	0.62–1.00	0.82	0.90	4-Hydroxy-3-methylbenzoic acid	136.0168
	5.59	159.1017	[M-H]-	6	0.58–0.99	0.73	0.80	2-hydroxy caprylic acid	135.0446
	5.83	514.2833	[M-H]-	2	0.50–0.95	0.64	0.80	Sulphated steroid	96.9582
	6.59	213.1488	[M-H]-	3	0.64–1.00	0.73	0.90	3-oxo-dodecanoic acid	No fragment
PTD with no BPD	4.04	415.1419	[M-H]-	3	0.59–1.00	0.73	1.00	Amino acid chain	No fragment
	4.81	383.1523	[M-H]-	2	0.67–1.00	0.82	0.80	3b,16a-Dihydroxyandrostenone sulfate	96.9582
	5.15	397.1316	[M-H]-	4	0.55–0.97	0.73	0.70	S-Adenosylmethionine	No fragment

RT = Retention Time; m/z = mass-to-charge ratio; ppm = part per million; AUC = Area under the Curve; ROC = receiver operating characteristics; Sp = specificity; Se = sensitivity; the optimal threshold used for specificity and sensitivity is the point closest to the top-left part of the plot with perfect sensitivity or specificity.

## Discussion

This study provides proof-of-concept evidence that the development of BPD is associated with a dysregulated metabolic profile of amniotic fluid, and it suggests that metabolic profiling of amniotic fluid could be a useful tool to differentiate preterm delivery from term delivery. To our knowledge, no previous untargeted study based on high-dimensional biology technique applied to amniotic fluid, has investigated the relationship between AF composition and the respiratory outcome in newborns.

BPD has traditionally been attributed to an arrest in the alveolar and vascular maturation of the developing lung[7,25] and to the injury of lung tissue inflicted by the combination of barotrauma and oxygen toxicity. Today BPD typically affects neonates born weighing less than 1000 grams and it is essentially a developmental disorder in which the immature lung fails to reach its full structural complexity. Accumulating evidence suggests that particular insults sustained during fetal life (intra-amniotic inflammation/infections, placental dysfunction) can lead to preterm delivery [26] and affect lung development before birth[8,27,28].

Thus far, some studies focused on the analysis of AF for the purpose of elucidating the relationship between prenatal factors, PTD and BPD have used a targeted analysis of a single or few mediators[3,10–13]. Using protein assays, for instance, some pro-inflammatory cytokines and other molecules, including interleukin-6 (IL-6), interferon-gamma-inducible protein (IP)-10[10], and matrix metalloproteinase (MMP)-8[11], appeared to be expressed at higher concentrations in the AF of women who subsequently delivered preterm. At the same time, high values of IL-6, IL-8, IL-1β, tumor necrosis factor (TNF)-α in the AF seemed to confer a higher risk for subsequent BPD[12,13]. Although each of these mediators provides useful information, our understanding of the complex pathogenetic mechanisms underlying preterm parturition syndrome and BPD may draw advantage from a more global approach, such as untargeted metabolomic analysis of amniotic fluid. Metabolomics consists in the analysis of low molecular weight metabolites created by cellular metabolic pathways through the use of mass spectrometry or nuclear magnetic resonance spectroscopy. This analytical approach is not driven by any a priori hypothesis, thus permitting metabolic patterns characteristic of a given pathological condition to be identified, and to eventually recognize potential biomarkers in the metabolic profile. As a result, new pathogenetic hypothesis may be formulated [29,30]. Appropriate statistical approaches are needed to extract information from the data set obtained. Specifically, multivariate methods have been introduced to integrate the results obtained by univariate statistical analysis. Unlike univariate analysis, multivariate statistical data analysis takes the correlation structure of the data collected into account, providing a holistic representation of the system under investigation. This brings to light synergic effects between variables that go undetected if one variable is considered at a time [31].

The risk of preterm birth was recently assessed in a retrospective study through the use of MS-based metabolomics applied on human AF [14]. The results indicated that metabolomic analysis on AF can be a novel approach to distinguish pregnancies with spontaneous preterm labor and intact membranes who will deliver at term from those who deliver preterm, irrespective of any intra-amniotic infection/inflammation. The present study extends these findings, showing that the two constrained PLS-DA models (one for each data set analyzed) were able to establish from the AF metabolic profile which pregnancies with preterm labor would end with a preterm delivery. This suggests that the metabolic pattern of the AF might be useful for predicting the risk of PTD in women with an episode of PTL. Interestingly among the key metabolites identified in patients who delivered preterm, we found increased concentrations of 4-hydroxy nonenal alkyne, supporting the role of oxidative stress in the preterm parturition syndrome [32].

Of potentially greater interest, our study suggests that the onset of BPD may be associated with a perturbed AF metabolic pattern during intra-uterine life. Indeed, the AF metabolome seems to be capable of distinguishing within those who delivered preterm, infants who will develop BPD and those who will not. This finding supports the hypothesis that BPD is determined not only by lung immaturity and postnatal factors (e.g. barotrauma and oxygen toxicity), but also by antenatal factors that impair the maternal-fetal equilibrium and the physiology of lung development[8,9].

As putative key metabolites for BPD development, we identified some hydroxylated and oxidated organic acids. We therefore suggest that AF collected in women whose offspring are bound to develop BPD is characterized by a particular fatty acids profile that may have a pathogenic role in the onset of BPD. The BPD group was characterized by reduced concentrations of a variable ascribable to S-adenosyl methionine, which is a methyl donor for biochemical methylation reactions and a precursor of the antioxidant glutathione. A reduction of this metabolite has been associated with increased oxidative stress [33,34]. This finding suggests that among premature babies those exposed to higher levels of in-utero oxidative stress are the most likely to develop BPD.We also found higher levels of a metabolite ascribable to DHEAS in the group of PTD without BPD than in the group of PTD with BPD. This finding confirms previous target studies demonstrating an association between reduced levels of cortisol and DHEAS, indicative of adrenocortical insufficiency, and BPD development [35,36]. Noteworthy, the agreement between our untargeted metabolomic approach and these previous target studies, supports the potential for metabolomics in identifying relevant metabolites associated with preterm delivery and BPD development. A limitation of this study is the lack of a validation cohort. This was a descriptive study conducted in a well-characterized set of patients with the aim of comparing the overall AF metabolic fingerprint of the recruited groups. Being a descriptive study, no external validation set was included in the design. The reliability of our findings is proved by internal validation obtained through full cross-validation and the Monte-Carlo sampling procedure. Nonetheless we recognize that further studies are necessary to replicate our findings in an independent cohort.

Another potential limitation of the study is the sample size. However, the possible interference of relevant clinical variables (metadata) on the classification of our limited number of samples has been excluded by applying an appropriate statistical strategy—the orthogonally Constrained PLS-DA [20]- which enabled us to infer that the group discrimination could be due only to the AF metabolite profile. In particular, the statistical data analysis permitted us to exclude that metabolic changes in the AF could be due to the different origin of samples, in according with a recent paper using ^1^H NMR-based metabolomic profiling [37].

Although our study is descriptive and preliminary in nature, it leads the way to the identification of patients at risk for preterm delivery, as well as those at risk for BPD, the most important complication of prematurity. Every intervention in medicine begins with prediction before we can test the effect of preventive or therapeutic strategies. The knowledge of a predictive metabolic profile, and possibly the identification of specific biomarkers of prediction, shines a light on the biology underlying preterm labor paving the way to the early identification of newborns at high risk of BPD, for whom target therapeutic measures might be developed. We recognize that, at this stage, we can only speculate on the metabolic nature of the discriminating compounds and that further studies are needed to fully characterize the biochemical structure of the metabolites that emerged.

## Conclusions

This study suggests that amniotic fluid metabolic profiling from mothers presenting with an episode of preterm labor may be a promising tool for identifying spontaneous preterm birth and fetuses at risk for developing BPD. Our findings strengthen the hypothesis that the injury responsible for BPD begins, at least partly, during the intra-uterine life. Further studies are required to validate the findings reported herein and understand the precise relationship between the differentially expressed metabolites and irreversible preterm parturition and the lung injury resulting in BPD.

## Supporting Information

S1 Fig **oCPLS2-DA model for PTD group versus TD group (positive data set);** A: score scatter plot after post-transformation of the model (PTD are reported as grey triangles while TD as open triangles); B: ROC curve of the model, calculated by 7-folds full cross-validation.
(TIF)

S2 Fig **oCPLS2-DA model for PTD with BPD versus PTD without BPD (positive data set);** A: score scatter plot of the model (PTD with BPD are reported as black triangles, PTD without BPD as grey triangles);B: ROC curve of the model, calculated by 7-folds full cross-validation.
(TIF)

S1 Material (DOC)

## Abbreviations

BPD: Bronchopulmonary Dysplasia
PTD: Preterm Delivery
TD: Term Delivery
PTL: Preterm Labor
AF: Amniotic Fluid
PROM: Premature rapture of membranes
oCPLS2-DA: Orthogonally Constrained Projection to Latent Structures-Discriminant Analysis
NHLBI: National Heart, Lung, and Blood Institute
CRP: C reactive protein; MS-Mass Spectrometry
UPLC: Ultra- Performance Liquid Chromatography
ESI: Electrospray source
PCA: Principal Component Analysis
PLS-DA: Projection to Latent Structures- Discriminant Analysis
RT: Retention Time
m/z: mass-to-charge ratio
AUC: Area Under the Curve
ROC: Receiver Operating Characteristics
ppm: part per million
Sp: Specificity
Se: Sensitivity
HMDB: Human Metabolome Database
BMI: Body Mass Index
BW: Birth weight
GA: Gestational age
SGA: Small for gestational age
DHEAS-3b,16a: Dihydroxyandrostenone sulfate
IL: interleukine
IP: inducible protein
MMP: matrix metalloptroteinase
TNF-αa: tumor necrosis factor α
