# Supplementary figures and images for: Untargeted Metabolomic Analysis of Amniotic Fluid in the Prediction of Preterm Delivery and Bronchopulmonary Dysplasia
Source: PLoS One. 2016 Oct 18;11(10):e0164211. doi: 10.1371/journal.pone.0164211 (PMC5068788; doi:10.1371/journal.pone.0164211)

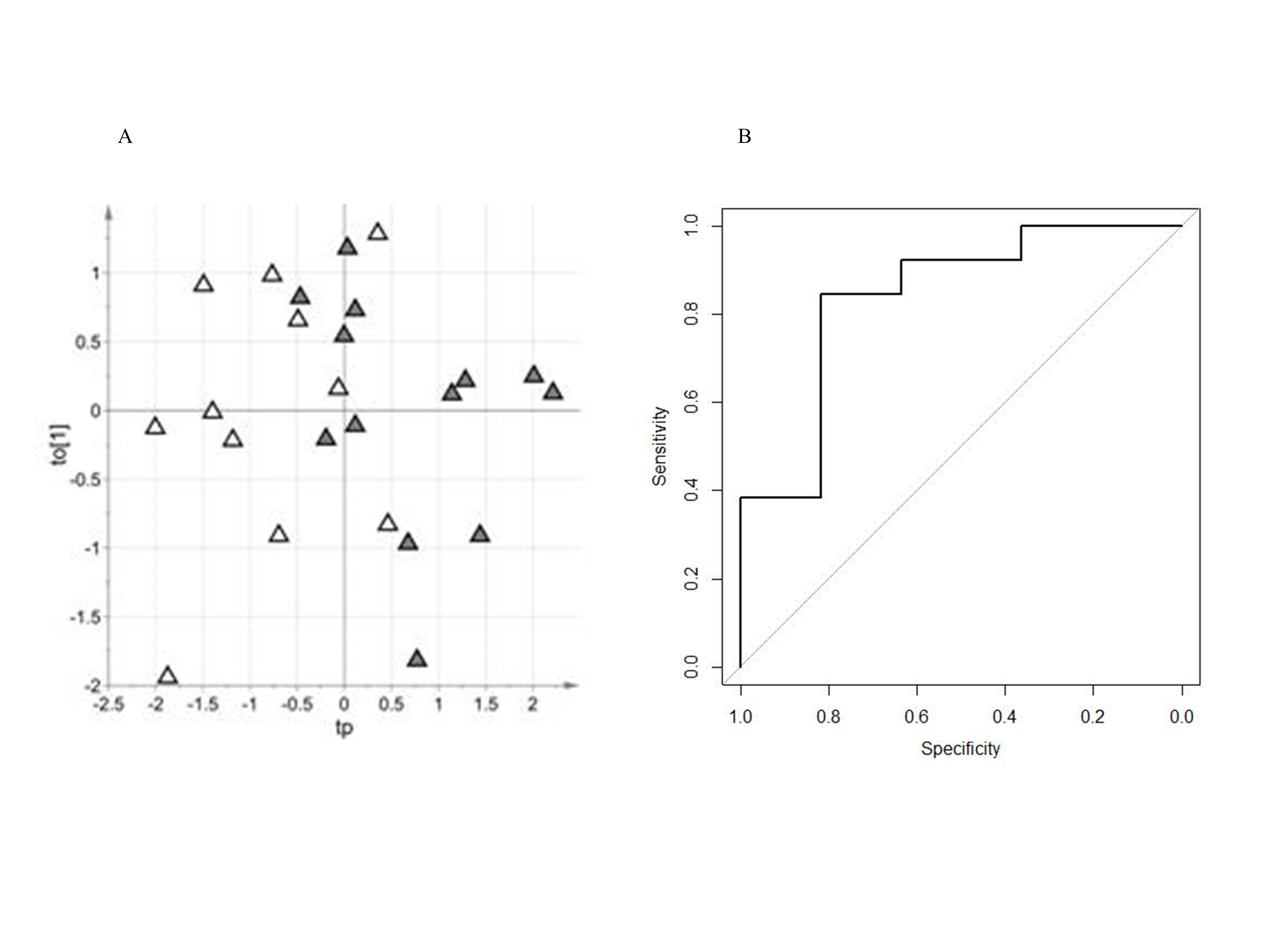

Supplement: S1 Fig — oCPLS2-DA model for PTD group versus TD group (positive data set); A: score scatter plot after post-transformation of the model (PTD are reported as grey triangles while TD as open triangles); B: ROC curve of the model, calculated by 7-folds full cross-validation. (TIF) [file pone.0164211.s001.tif]

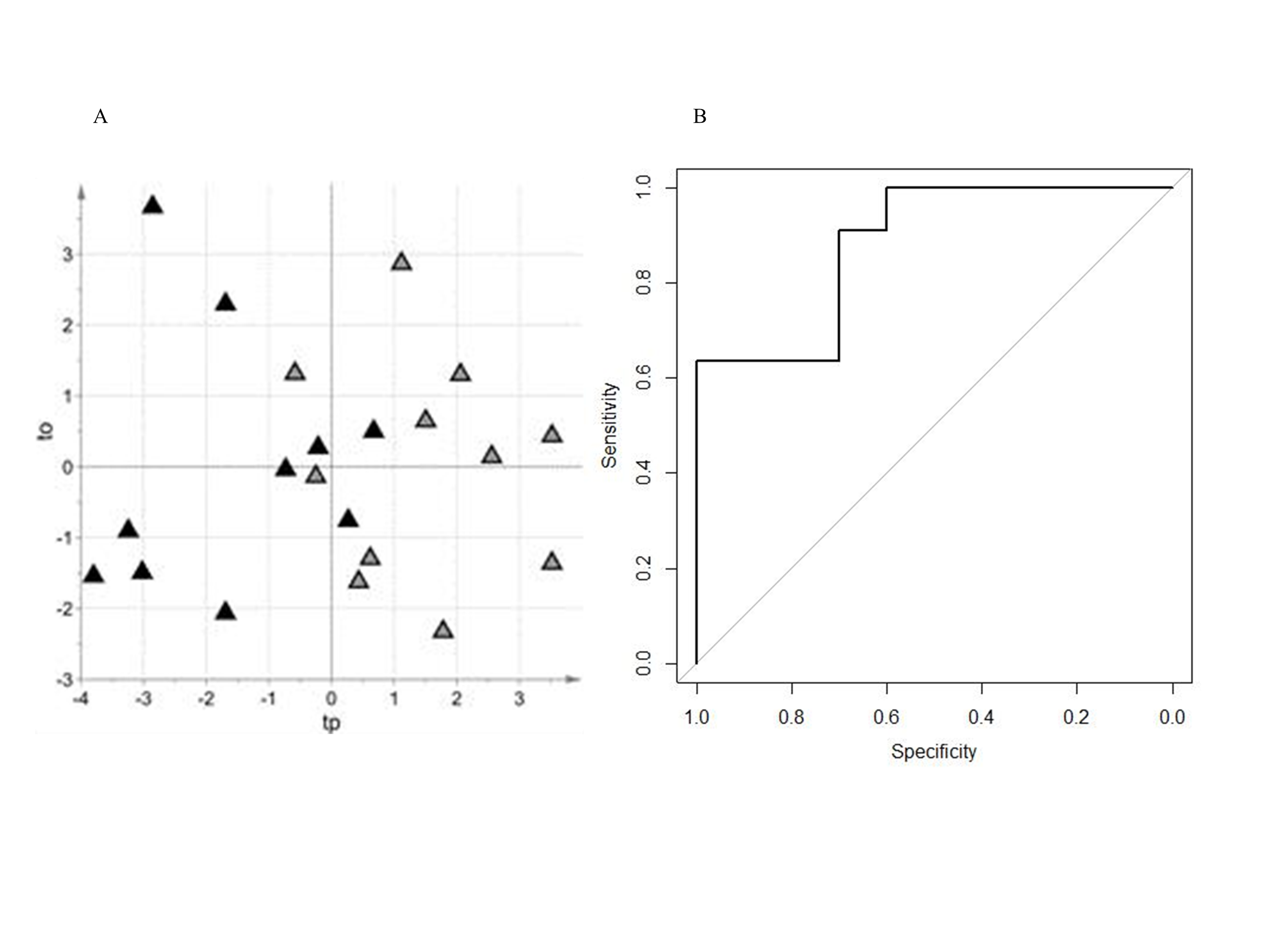

Supplement: S2 Fig — oCPLS2-DA model for PTD with BPD versus PTD without BPD (positive data set); A: score scatter plot of the model (PTD with BPD are reported as black triangles, PTD without BPD as grey triangles);B: ROC curve of the model, calculated by 7-folds full cross-validation. (TIF) [file pone.0164211.s002.tif]
