# Supplementary material for: Untargeted Metabolomic Analysis of Amniotic Fluid in the Prediction of Preterm Delivery and Bronchopulmonary Dysplasia
Source: PLoS One. 2016 Oct 18;11(10):e0164211. doi: 10.1371/journal.pone.0164211 (PMC5068788; doi:10.1371/journal.pone.0164211)
Supplement: S1 Material — (DOC) [file pone.0164211.s003.doc]

**Online S1 Supplementary Material**

**Untargeted Metabolomic Analysis of Amniotic Fluid in the Prediction of Preterm Delivery and Bronchopulmonary Dysplasia**

Eugenio Baraldi^1,2*^ , Giuseppe Giordano^1,2^ , Matteo Stocchero^3^ , Laura Moschino^1,2^ , Patrizia Zaramella^1^ , Maria Rosa Tran^1^, Silvia Carraro^1,2^ , Roberto Romero^4^ , Maria Teresa Gervasi^1^

^1^Department of Women’s and Children’s Health, University of Padova, Padova, Italy

^2^Città della Speranza Institute of Pediatric Research (IRP), Padova. Italy

^3^S-IN Soluzioni Informatiche, Vicenza, Italy

^4^Perinatology Research Branch, NICHD, NIH, DHHS, Wayne State University/Hutzel Women's Hospital, Detroit, USA.

* Corresponding author:

E-mail: [eugenio.baraldi@unipd.it](mailto:eugenio.baraldi@unipd.it) (EB)

**Study design and population**

We conducted a study on 32 infants born from 32 mothers who had undergone an amniocentesis between 21 and 28 gestational weeks because of spontaneous preterm labor (due to PROM, chorioamnionitis, flow alterations or other causes). Amniocentesis had been performed at the participating institutions (Padova and Treviso general hospitals, Veneto region, Italy) to assess the microbial state of the amniotic cavity and to diagnose intra-amniotic infection/inflammation[1]. Twins and newborns with congenital anomalies were excluded. The amniotic fluid samples were collected by the same physician (MTG) with a standardized procedure.

Twenty-four of the 32 AF samples were obtained from trans-abdominal amniocentesis, the other 8 by amniocentesis at the time of cesarean delivery. Five milliliters of AF were collected, frozen and stored at - 80° until the time of the analysis.

For the purposes of our data analysis the samples were considered as follows:

1. In the first step, we aimed to assess whether preterm delivery could be discriminated by the metabolomic profile of amniotic fluid, by focusing on samples (n=24/32) collected at least 1 day before birth. Among these, the metabolomic profile was compared with those of patients delivered preterm (PTD group, n=13/24) and those of term newborns (TD group, n=11/24).
2. The second step of the study consisted of determining whether amniotic fluid analysis could discriminate infants bound to develop BPD. Twenty-one samples from pregnancies which resulted in a preterm delivery (n=21/32) were analyzed according to whether infants developed BPD (PTD with BPD, n=10/21; PTD with no BPD, n=11/21).

The study was approved by the Institutional Review Boards of the participating Institutions (Comitato Etico per la Sperimentazione, Padova and Treviso General Hospitals, protocol number 24139, Veneto Region, Italy). All the women gave their written informed consent to their AF being used for research purposes.

**Amniotic fluid metabolite analysis by Mass Spectrometry (MS) combined with Ultra- Performance Liquid Chromatography (UPLC)**

## Sample preparation

At the time of the analysis, the amniotic fluid samples were thawed and mixed with a vortex. A 420 µL aliquot of each sample was diluted with methanol (conserved in ice at 0°C) in a ratio of 1:6, in order to deproteinize the fluid and to avoid formation of protein precipitate. Aliquots were put at -20°C for 30 minutes to facilitate the deproteinization process. After centrifugation at 6000 g for 15 minutes at a temperature of +4°C, the supernatant was separated and transferred into Falcon tubes, which were placed in a centrifugal vacuum concentrator (Centrivap Concentrator, LABCONCO) at room temperature for approximately 12 hours. This process enabled the solution to be dried by sublimation and further concentrated [2,3]. After evaporation, samples were then re-suspended with 210 µL of H_2_O and 0,1% of formic acid, centrifuged at 16000g, and transferred to vials.

## Chromatographic analysis and mass spectrometry

The metabolic analysis of the AF samples was performed with a Q-ToF Synapt G2 (Waters) high resolution mass spectrometer interfaced with a UPLC (Ultra Performance Liquid Chromatography) system (Acquity-Waters). LC-MS ULTRA grade solvents and reagents (Sigma St. Luis, MO, USA) and water type MilliQ with 1 grade of purity were used for the analysis.

The chromatographic analysis was performed through the reverse-phase HSS T3 1.8 µm, 2.1 mm and 100 mm column (Acquity HSS T3, Waters co., Miliford, MA USA) at 40°C. The gradient used to analyze eluent A was H_2_O and 0,1% of formic acid, and for eluent B it was methanol:acetonitrile 90:10 and 0,1% formic acid. The percentage of eluent B varied over time as follows: 5% at the baseline; 30% at 3.5 minutes; 95% at 6.5 minutes; 5% at 11 minutes; with a constant flow of 500 µL/min. MS analysis was conducted with an Electrospray source (ESI) in both positive (ESI+) and negative (ESI-) ionization mode. The ESI settings were: a capillary voltage of 3 KV in the positive polarity and 1.5 KV in the negative; a source temperature of 110°C; a desolvation temperature of 350°C; and a nitrogen gas flow of 600 L/h. The mass scan was set in a range between 20 and 1200 amu in 0.3 sec. Two examples of chromatographic profiles are shown in Fig 1. All the samples were injected in triplicate using random sequences to prevent any analytical artifacts from conditioning the samples’ classification.

In order to assess analytical reproducibility during chromatographic analysis, quality control (QC) samples consisting of 20µL of each AF sample, were injected every ten injections. The variation in the QC intensity (from the first to the last) on the positive and negative sequence had a mean value of less than 8% and less than 5%, respectively. A standard mix of known compounds (Sulfadimethoxine, Val-Tyr-Val, Leucine-Enkephalin, Erithromycin and Caffeine) was analyzed in the sequence to verify mass accuracy and process efficiency during the analytical process. AF samples were analyzed in both LC-MS and LC-MS^E^ for the structural hypothesis; the letter is a non-selective fragmentation technique based on the passage of ionized molecules through the collision cell at High and Low collision energy during the chromatography.

The loss in sensitivity, expressed as CV% of each standard intensity (as Integrated Area) in the repeated runs of standard samples had a mean value of 7.8% and 4.4% for the positive and negative datasets, respectively.

The mass accuracy was ≤ 3 ppm for all compounds of the standard samples (0.99±1.07ppm).

**Data processing and pre-treatment**

The UPLC-MS data were transformed into data tables using the MarkerLynx software (Waters co., Miliford, MA USA). The parameters used for data extraction were optimized by means of the preliminary analysis of the QC samples, which generated the RT_mass variables (where RT is the retention time and mass the m/z ratio of the chemical compound). For the 3 replicates of each sample, variables were calculated in terms of median mass intensity. The application of two ionization modes (positive and negative) led to the generation of two data sets.

Variables with more than 20% of the data missing within each group were rejected to avoid generation of spurious statistical models including unrealistic combinations of the variables being measured. For each variable passing said filter, the minimum value measured for the variable was used to replace any missing data. A Median Fold Change normalization was applied to take dilution effects into account, while log-transformation was performed to reduce the scale effects of the variables on the models. The resulting data sets were mean centered before conducting statistical data analysis.

**Statistical data analysis**

In the study, to exclude any confounding effects with respect to the clinical groups under investigation, we applied a new version of PLS-DA to the data sets generated by UPLC-MS that enables orthogonal constraints to be included in the latent variable calculation. We called this statistical method ‘orthogonally Constrained PLS-DA’ (oCPLS2-DA)[4]. Given the limited number of subjects included in the study and the large number of variables used to represent their metabolome, confounding effects between the clinical groups under investigation and clinical data not closely related to the factors being investigated might be included in the statistical models and give rise to false-positive results. In this respect, the crucial clinical data were used to construct the matrix, specifying the constraints of the model, and thus removing information related to the metadata from the latent structure revealed by the discriminant model. We describe in the following the theoretical framework underlying oCPLS2-DA.

We consider a matrix and we want to calculate a weight vector able to project the X-block as in the framework of the PLS [5] algorithm under the constraint . The maximisation problem at the iteration of the PLS algorithm can be formulated as

A1

where and are the residual matrices for the X- and the Y-block, respectively, and is the weight vector for projecting the Y-block.

The vector must belong to the kernel of or to be orthogonal to the column space of . We chose the second route by assuming

where is the orthogonal projection matrix able to transform each vector into a vector orthogonal to . Indeed, we can calculate

.

Then, the maximisation problem A1 can be re-written as

.

and the solution obtained by applying the Lagrange’s multipliers method. The weight vector to use results to be the eigenvector corresponding to the highest eigenvalue of the problem

A2

where .

This result can be usefully applied to find score vectors orthogonal to the column space of a matrix for performing constrained PLS regression. The maximisation problem at the iteration of the iterative algorithm for PLS can be now formulated as

.

It can be proved that at any iteration. As a consequence, the solution is A2 having

where is obtained by singular value decomposition of .

The following algorithm is able to calculate the orthogonally constrained PLS model for given , and matrices

*i* = 1

1

*i* = *i* + 1 go to 1 for other components

where . After *A* iterations, the regression model

can be obtained by calculating the regression coefficient matrix

.

The method introduced in Reference 5 to rotate the PLS solution in order to split the score space into orthogonal and parallel scores can be applied to orthogonally Constrained PLS with the result to obtain a latent space that can be more easily investigated. Orthogonally Constrained PLS can be used to drive discrimination by introducing suitable dummy variables for the response matrix according to PLS-DA technique.

**Identification of relevant variables**

The METLIN database provided the most useful information for characterization of the selected variables. The search was run with the following parameters: m/z ratio of the variable analyzed; charge of the mass (depending on the ionization mode); ionization type [M+H]^+^ or [M+H]^-^ (as electrospray ionization is a soft technique which usually produces single charged molecules); error tolerance of less than 5 ppm (where ppm = ((M_measured_ – M_expected_) / M_expected_)*10^6^). In order to obtain additional structural information, the metabolites were analyzed in terms of their molecular ion to establish their elemental composition, and of their MS^E^ fragmentation spectra (using the Mass Fragment tools in the Masslynx software) to confirm their hypothetical structure. This approach has helped us to establish the chemical structure of the hypothetical metabolite derived from the initial analysis.

S1 Fig ~~S1~~. oCPLS2-DA model for PTD group versus TD group (positive data set); A: score scatter plot after post-transformation of the model (PTD are reported as grey triangles while TD as open triangles); B: ROC curve of the model, calculated by 7-folds full cross-validation.

**S2 Fig ~~S2~~.** **oCPLS2-DA model for PTD with BPD versus PTD without BPD (positive data set);** A: score scatter plot of the model (PTD with BPD are reported as black triangles, PTD without BPD as grey triangles);B: ROC curve of the model, calculated by 7-folds full cross-validation.

**References**

1. American College of Obstetricians and Gynecologists; Committee on Practice Bulletins-Obstetrics: ACOG practice bulletin n° 43: Management of preterm labor. Obstet Gynecol. 2003;101:1039-47.
2. Yin P, Mohemaiti P, Chen J, Zhao X, Lu X, Yimiti A, Upur H, Xu G. Serum metabolic profiling of abnormal savda by liquid chromatography/mass spectrometry. J Chromatogr B Analyt Technol Biomed Life Sci. 2008;871:322-7.
3. Bruce SJ, Tavazzi I, Parisod V, Rezzi S, Kochhar S, Guy PA. Investigation of human blood plasma sample preparation for performing metabolomics using ultrahigh performance liquid chromatography/mass spectrometry. Anal Chem. 2009;81:3285-96.
4. Anesi A, Stocchero M, Dal Santo S, Commisso M, Zenoni S, Ceoldo S, et al. Towards a scientific interpretation of the terroir concept: plasticity of the grape berry metabolome. BMC Plant Biology 2015;15:191.
5. Stocchero M, Paris D. Post-transformation of PLS2 (ptPLS2) by orthogonal matrix: a new approach for generating predictive and orthogonal latent variables. J. Chemometrics DOI: 10.1002/cem.2780.
